# Carotid Pulse Wave Analysis: Future Direction of Hemodynamic and Cardiovascular Risk Assessment

**DOI:** 10.31662/jmaj.2020-0108

**Published:** 2021-04-02

**Authors:** Sam Parittotokkaporn, Denis de Castro, Andrew Lowe, Romana Pylypchuk

**Affiliations:** 1 School of Medical Sciences, Faculty of Medical and Health Sciences, University of Auckland, Auckland, New Zealand; 2 Biomedical Consulting, Paris, France and Auckland, New Zealand; 3 Institute of Biomedical Technologies, Auckland University of Technology, Auckland, New Zealand; 4 School of Population Health, Faculty of Medical and Health Sciences, University of Auckland, Auckland, New Zealand

**Keywords:** cardiovascular diseases, atherosclerosis, arterial stiffness, carotid artery, pulse wave analysis, wearable sensors

## Abstract

Evaluation of the hemodynamic function of the cardiovascular system *via* measurement of the mechanical properties of the large arteries may provide a substantial improvement over present techniques. Practitioners are familiar with the problem of low reproducibility of conventional sphygmomanometry, which exhibits reasonable accuracy but low precision owing to its marked variability over time and in different circumstances (e.g., the white coat effect). Arterial wall stiffness is a consequence of atherosclerosis developing over time; thus, it has little short-term variability and is thus preferable to be used as a prognostic marker. In particular, arterial stiffness can be evaluated at the carotid artery using noninvasive approaches based on wearable sensor technologies for pulse wave analysis. These enable the assessment of central pressures and pulse waveform parameters that are expected to replace peripheral blood pressure measurement using the inflatable cuff. In this study, we discuss this simple and inexpensive technique, which has been shown to be reliable with the clinical and epidemiological evidence for its use as a biomarker of cardiovascular risk.

## Introduction

**Figure 1.**
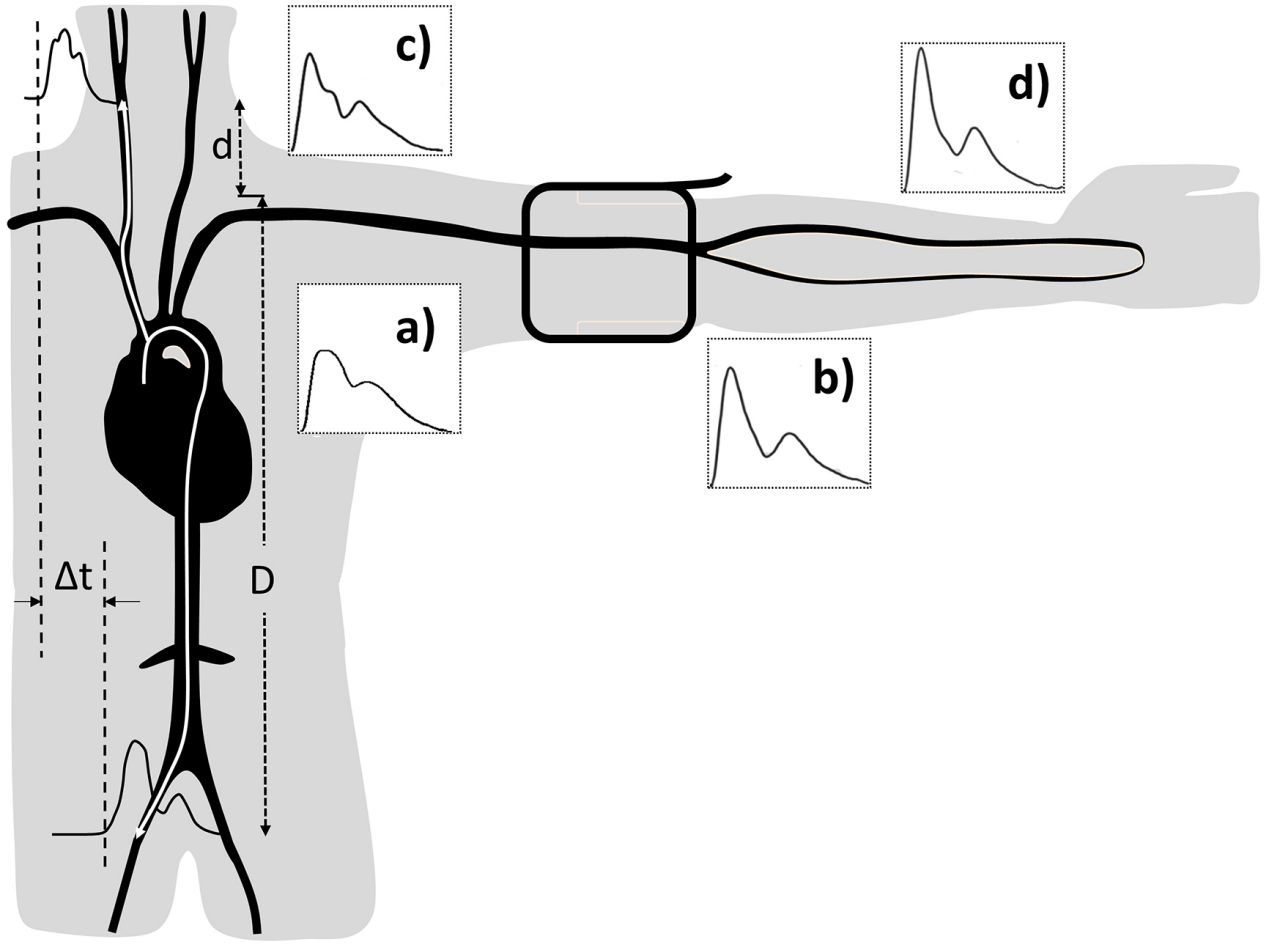
Amplification of the pressure waveform moving from the aorta (a) to the brachial (b), carotid, (c) and radial arteries (d). cf-PWV or aortic PWV is a ratio of the distance D-d and the transit time Δt between the carotid and femoral arteries.

Cardiovascular diseases (CVDs), such as stroke and heart attack, are the major cause of mortality in economically developed countries, accounting for about 30% of deaths annually ^(1)^. The majority of CVDs is related to risk factors such as advancing age, high blood pressure (BP), obesity, lack of physical activity, smoking, and diabetes. All of the risk factors interfere with the blood supply to vital organs due to the development of arterial stiffness and arteriosclerosis in the arterial system. There is a scientific evidence proving that arterial stiffness assessed from the carotid artery is a standard method and independent predictor of cardiovascular events ^(2)^. Furthermore, assessment at the carotid artery has been accepted as a better predictor of cardiovascular events compared with measurement at the peripheral artery obtained from conventional cuff BP devices ^(3)^. Cuff sphygmomanometry has been used for more than 100 years for recording BP at the upper arm in general medical practice. It exhibits reasonable accuracy but low precision owing to its marked variability over time and in different circumstances (e.g., the white coat effect) ^(4)^. In fact, brachial artery BP imprecisely measures two limit values of systolic and diastolic BPs regardless of the true representation of the BP profile in the entire cardiac cycle. This method only allows a BP measurement at the peripheral artery which does not reflect the real hemodynamics (Figure 1). Thus, the central pulse pressure and its determinants are established as better predictors of CVD risk than conventional brachial artery assessment ^(5)^.

There are numerous methods to evaluate the function of the carotid artery. One of the noninvasive approaches is pulse wave analysis (PWA), which provides useful information regarding the mechanical properties and hemodynamic function of the carotid artery. However, there is a small number of clinical research studies on how to interpret and use PWA data of the carotid artery in clinical practice. This review presents an approach to the clinical application of PWA of the carotid artery and determines how to analyze carotid pulse waveform using noninvasive techniques and how to measure carotid arterial stiffness in combination with other parameters related to cardiovascular events.

## Clinical Significance of the Carotid Artery

Carotid arteries are the major arteries in the neck that supply oxygenated blood from the heart to the brain. The average diameters of the common carotid artery in adult males and females are 6.5 and 6.1 mm, respectively ^(6)^. The two internal carotid arteries contribute 80% of the blood supply to the brain, whereas the external carotid arteries deliver blood to the head and neck ^(7)^. The internal carotid artery provides high blood flow exceeding 238.8 mL/min ^(8)^, whereas the brachial artery, which is a common place of cuff BP, provides only 72.7 mL/min on average ^(9)^. The pathological changes of the carotid artery can affect the brain, and the hemodynamic changes at the heart, aorta, and brain may be detected at the carotid artery. For example, the severe narrowing of the carotid artery can block blood flow, or a piece of atherosclerotic plaque can break off and obstruct an artery inside the brain, which results in stroke. Hence, there is an evidence that a cardiovascular event is more closely related to the carotid artery rather than the brachial artery ^(5)^. For practical applications, the brachial or radial artery waveform is more readily recorded with more consistent reproducibility and repeatability with less prone to noise introduced by incorrect vessel applanation or respiration as can occur with carotid waveforms ^(10)^. However, the disadvantages of the carotid pulse measurement can be solved using new technologies of wearable devices and contactless techniques, including analytical algorithms. Carotid PWA is a simple, valid, reliable, noninvasive, and inexpensive technique, which offers a great clinical and epidemiological potential ^(11)^. Therefore, research is more focused on the hemodynamic measurement of the carotid artery rather than the peripheral arteries with the noninvasive determination of pressure waveform ^(12)^.

## Methods to Measure Pulse Waveform

The methods of pulse wave assessments at the carotid artery can be invasive and noninvasive. In this review, only the latter will be described. The technique of noninvasive PWA depends on different principles and the types of pulse wave. In clinical practice, PWA is commonly used by the hand-held tonometry probe. It is simple to use, noninvasive, and accurate and also utilizes a small strain gauge sensor that detects the force on the arterial wall ^(13)^. The principle of applanation tonometry involves a partial compression of a pulsating carotid artery against the muscle and vertebral body of the neck that allows a pulse wave spreading in the skin impacting the sensor.

One commonly used noninvasive method for obtaining the pulse waveform is photoplethysmography, which involves the placement of an optical sensor over the artery. This method is based on the same principle as pulse oximeters to measure the blood volume waveform. In general, the obtained waveform represents the volume of oxygenated blood perfusion around the tissue supplied by the branches of the artery under the sensors. This volume pulse wave has similarities with pressure pulse waveform ^(14)^. Doppler ultrasound is a noninvasive medical image scanner. It is a standard tool for the examination of the carotid artery. It can quantify the extent of arterial layer thickening and atherosclerotic plaque formation as well as evaluate the blood flow to estimate the degree of stenosis. One of the great benefits of ultrasound is its ability to measure wall thickness as delicate as the innermost layer of the artery. This intima-media thickness of the carotid artery is also the surrogate marker to evaluate the risk of cardiovascular events ^(15)^. However, Doppler ultrasound is highly operator-dependent, time-consuming, and expensive.

## Pulse Wave Analysis

**Figure 2.**
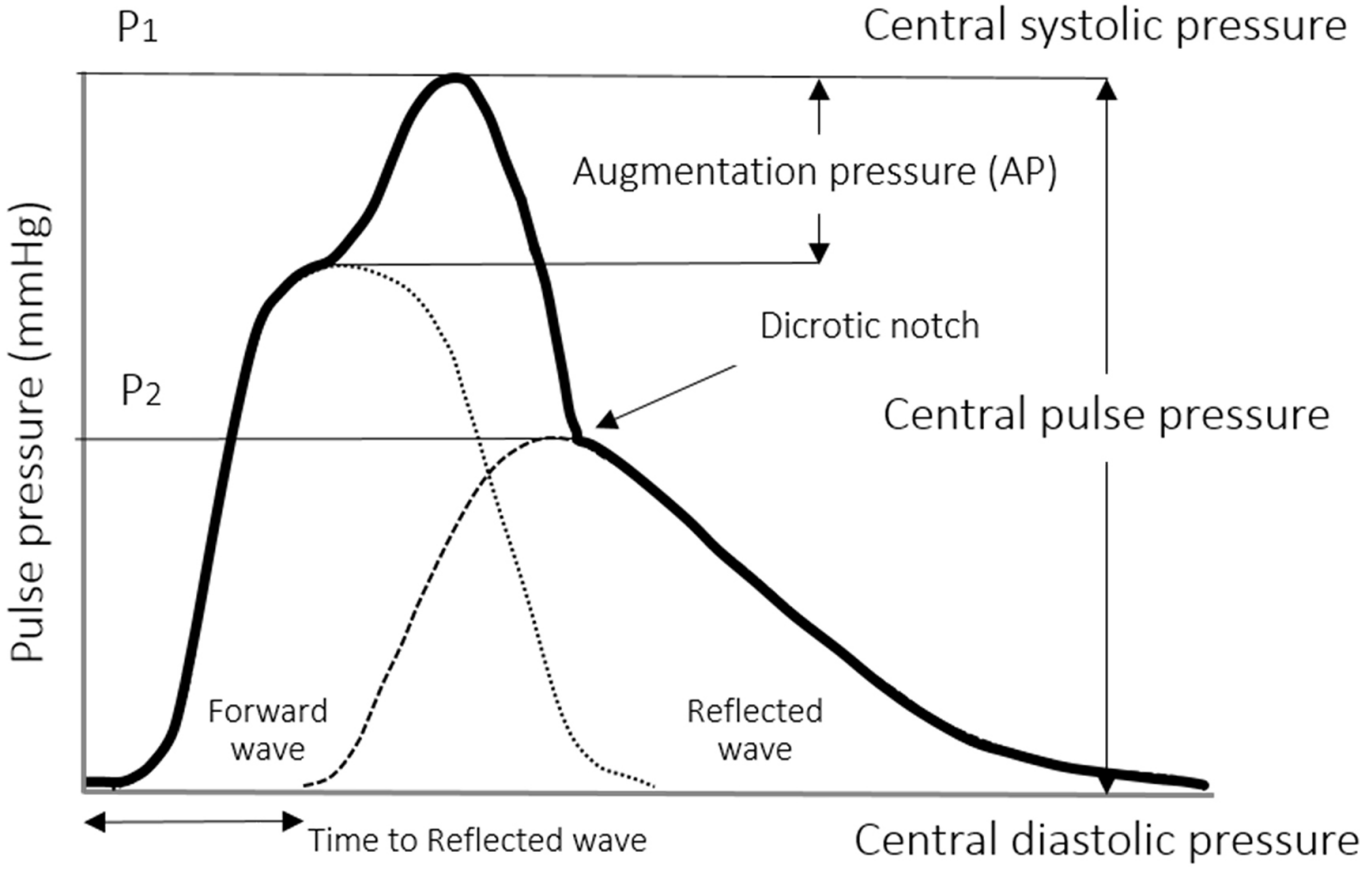
Analysis of central pulse pressure waveform.

Pulse waves are generated by heart contraction and arterial wall distensibility. Arteries distend in response to the increase in BP and volume caused by the contraction of the left ventricle. During heart relaxation, the arteries contract to release the elastic energy accumulated during distension. Therefore, the arteries produce the pulse following the cardiac cycle and propagate energy in the form of pulse waves. Pulse waves produce rhythmic changes in BP and flow, which can be studied as pressure and velocity waveforms ^(16)^. The pressure pulse waveform is divided into the systolic and diastolic part. The systolic component mainly arises from a forward pressure wave, whereas the diastolic component arises from reflected pressure waves ^(17)^. This contour of the central pressure pulse in Figure 2 is typical for the arteries close to the heart, e.g., carotid artery.

The pulse pressure waveform is the sum of the forward pressure wave generated by the heart contraction and the reflected pressure waves that resulted from wave terminations in the peripheral arterial trees ^(18)^. An early return to the heart of the reflected waves is caused by stiff arteries that increase the speed of the traveling pressure waves and change the pressure waveform by increasing the central systolic and pulse pressures.

In the carotid arteries, compared with the peripheral arteries, the pressure changes are slightly different from those in the aorta. While the pressure waveform travels from the aorta to the peripheral arteries, the pulse pressure increases due to the amplification resulting from the continuous changes of the systolic pressure throughout the arterial tree (Figure 1). The systolic pressure may be up to 40 mmHg in the brachial artery, which is higher than that in the aorta ^(19)^. Research has been conducted to determine carotid pressure as a surrogate of aortic pressure, i.e., the central pressure ^(20)^. Pulse pressure amplification increases with the increase in arterial stiffness as both the forward and the reflected waves propagate more rapidly along the stiff vessels than the normal ones.

Augmentation pressure is the additional aortic systolic pressure produced by the return of the reflected waves at the central aorta ^(21)^. Hence, the augmentation index (AIx) is the augmentation pressure as a percentage of the central pulse pressure. It is a composite measure of arterial wave reflection and systemic arterial stiffness ^(21)^. In practice, the AIx is used for the expression of the increase in intra-arterial pressure caused by the reflected wave. The augmentation index is typically measured as the difference of the first systolic (P1) and second pressure peak (P2), which is represented as a percentage of the pulse pressure (PP); AIx (%) = (P1 - P2) × 100/PP ^(22)^. Increased AIx of the carotid artery caused by the increase in the aortic pressure will thus provide an estimate of the stiffness of the arterial system in its complexity. AIx can be evaluated either for the central AIx of the aorta and peripheral AIx of other distal arteries ^(23)^. The augmentation index has been shown to be a predictor of adverse cardiovascular events in a variety of patient populations ^(24)^. A high augmentation index is associated with numerous vascular conditions as presented in Table 1.

**Table 1.** The Role of the PWA as an Additional Method for Evaluating Pathophysiological Conditions.

Conditions	Events	Mechanisms	Measurements
Carotid artery diseases	Intima-media thickness ^(43)^	Atherosclerosis	cf-PWV
	Carotid stenosis ^(33)^	Narrowing of artery	PWTT
Cerebrovascular diseases	Ischemic stroke ^(44)^	Emboli	cf-PWV
	Hemorrhagic stroke ^(45)^	High BP	cf-PWV
	Cerebral aneurysm ^(46)^	Arterial wall damage	cf-PWV, AIx, CPP
	Microvascular disease ^(47)^	Arterial stiffness	cf-PWV
	Migraine ^(48)^	Vasodilatation	cf-PWV, AIx
Heart diseases	Coronary heart disease ^(49)^	Atherosclerosis	cf-PWV
	Chronic heart failure ^(50)^	High PVR	cf-PWV, AIx, PP
	Arrhythmia ^(51)^	Atrial fibrillation	cf-PWV, AIx, CPP
	Aortic valve disease ^(52)^	Aortic stenosis	cf-PWV
Systemic diseases	Hypertension ^(53)^	Arterial stiffness	cf-PWV
	Diabetes ^(54)^	Endothelial dysfunction	cf-PWV, AIx
	Hypercholesterolemia ^(55)^	Endothelial dysfunction	cf-PWV, c-PWV
	Chronic kidney disease ^(56)^	Microcirculation	cf-PWV, AIx
	Abdominal aortic aneurysm ^(57)^	Arterial wall damages	cf-PWV, AIx

AIx = augmentation index; cf-PWV = carotid-femoral pulse wave velocity; c-PWV = local carotid PWV; CPP = central pulse pressure; PP = pulse pressure; PVR = peripheral vascular resistance; PWTT = pulse wave transit time.

The speed of a pulse in the circulatory system is pulse wave velocity (PWV) which occurs when a pressure pulse generated by heart propels blood stream along the vascular tree. PWV is influenced by the geometric and elastic properties of the arterial wall and increases in proportion with arterial stiffness. This can be explained through the calculation of PWV using the Moens-Korteweg equation; *PWV=√Eh/2rρ*, which relates PWV to a measure of stiffness of an elastic artery as Young’s elastic modulus (E), the arterial wall thickness (h), the end-diastolic radius of the artery (r), and the blood density (ρ) ^(25)^. PWV represents the speed by which a pressure pulse travels from the root of the aorta to the peripheral arteries. PWV can be assessed *via* the pulse pressure wave at two different locations along the arteries (e.g., carotid and femoral arteries) of the body using oscillometric, tonometric, volume plethysmographic, and photoplethysmographic devices ^(11)^. To calculate PWV, the travel distance can be simply divided by the transit time as presented in Figure 1.

Arterial distensibility and compliance are related to the elastic properties of the arterial**wall. Arterial distensibility is a measure of the arterial ability to expand and contract with pulse wave propagation during heart contraction. An increase in the arterial wall stiffness causes a decrease in arterial distensibility. Thus, arterial compliance is a ratio between arterial volume change (ΔV) and pressure change (ΔP). Hence, an increase in blood volume occurs in a vessel with increasing pressure as a proportion to arterial compliance and elasticity ^(8)^. A large artery has a higher compliance value than a small artery which demonstrates very small arterial volume changes against large arterial pressure change ^(9)^. Therefore, the small artery (e.g., radial artery) with the muscular component in the peripheral vascular system has a lower compliance value than the large artery (e.g., carotid artery) with the main elastic component in the central vascular system.

## Clinical Measurements of PWA of the Carotid Artery

**Figure 3.**
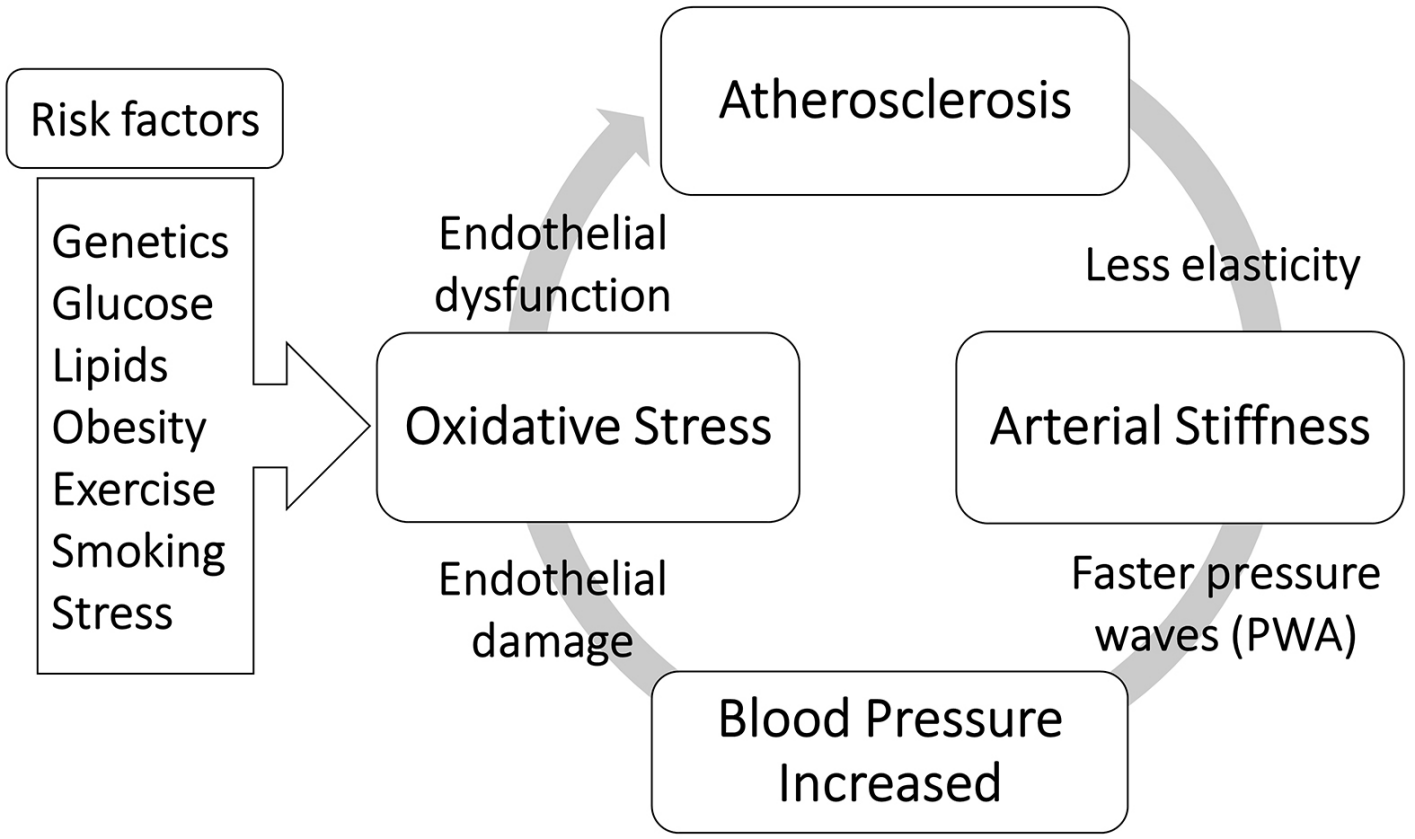
Cycle of increasing blood pressure due to structural and functional vascular changes.

Over the last few decades, attention to the dynamical measurement of pulse waveform has increased due to the understanding of the relationship between the specific changes of waveform parameters and the structural and functional vascular changes as a consequence of atherosclerosis and arterial stiffness and other pathological conditions. Recently, endothelial dysfunction has become the most mentioned atherogenesis development. Multivariable risk factors cause inflammation of the endothelial layer of the blood vessels, which leads to the stiffness of the arteries in which elastic fibers are reduced. As a result, peripheral vascular resistance and BP increase. Therefore, high blood flow can deteriorate the function of the endothelium after a repeated cycle of atherosclerosis progression, as presented in Figure 3. The carotid artery has a dampening and conduit function through which blood is pumped from the heart to the brain. Therefore, the pathological conditions of the carotid artery can lead to alterations in central BP and pulse waveform measurements as described in the next section.

### Carotid atherosclerosis

Atherosclerosis is a systemic vascular disease, but it can predominantly affect the local arteries of specific organs, such as the cerebral artery, coronary artery, and renal artery. In fact, atherosclerosis is the most common cause of carotid artery diseases that causes various degrees of conditions, from carotid stiffness to severe carotid stenosis. Therefore, carotid atherosclerosis might be related to a wide range of systemic arterial changes within the central and peripheral circulatory system. These changes affect the BP augmentation causing higher systolic pressures, widening pulse pressure, increasing vascular resistance, and resulting in left ventricular hypertrophy. Consequently, the risk of stroke and renal impairment increases, as the blood vessels of the brain and kidneys are exposed to great pressure fluctuations ^(26)^.

### Carotid stiffness

Carotid arterial stiffness occurs as a consequence of physiological and pathological changes, such as aging and atherosclerosis. The Rotterdam Study conducted on 3,000 elderly subjects reveals that arterial stiffness is strongly associated with atherosclerosis at various sites in the cardiovascular system ^(27)^. The parameters that describe arterial stiffness include compliance, which is the ability of the arterial wall to become stretched ^(28)^. The consequence of carotid stiffness is an increased amplitude of the reflected wave and PWV, which is greatly important and commonly used for the measurement of systemic arterial stiffness. Therefore, systolic BP increases, which causes an increase in the left ventricular workload and subsequent ventricular hypertrophy, whereas diastolic BP decreases, which leads to a decrease in coronary perfusion ^(29)^. As a result, evaluation of local carotid stiffness using echo-tracking devices is an indirect assessment of coronary atherosclerosis as well as a predictor of cardiovascular mortality in end-stage renal disease ^(28)^.

### Carotid stenosis

Carotid stenosis is defined as the atherosclerotic narrowing of the proximal internal carotid artery. Stenosis grading is divided into 50%-70% as moderate and exceeding 70% diameter narrowing as severe ^(30)^. Physiologically, arterial stenosis reduces blood flow and generates turbulent flow, which can auscultate as a bruit during a clinical examination. However, McColgan et al. found that the presence of carotid bruit does not indicate carotid artery stenosis and is not correlated with the degree of narrowing, whereas the absence of carotid bruit can be a specific sign to rule out carotid stenosis ^(31)^. In the general population, the most commonly used screening test for carotid stenosis is ultrasonography. However, it has proven to yield many false positives in patients, which can lead to misdiagnosis and unnecessary treatments ^(32)^. The measurement of pulse wave transit time and PWV might be an alternative method for providing a simple and reproducible noninvasive technique for the evaluation of varying degrees of carotid artery stenosis ^(33)^.

## Effects of Pathological Phenomena on the PWA

### Hypertension

Substantial research evidence suggests that central BP measured from the aorta and carotid artery is better related to the risk of cardiovascular events than brachial pressure ^(5)^. Vaccarino et al. reported that the increase in central pulse pressure by about 10 mmHg increases the risk of heart failure by about 14%, coronary heart disease by about 12%, and mortality by about 6% in the population older than 65 years ^(34)^. To monitor the hypertensive condition, the central BP values, i.e., systolic and diastolic BP and augmentation index, are used as gold standard parameters. PWV is also a useful marker of target organ damage, such as the heart, brain, aorta, and kidney, in hypertensive patients ^(35)^. In addition, decisions based on central pressure obtained from the carotid artery, rather than brachial pressure, are more likely to be adequate for the diagnosis and proper management of hypertension as anti-hypertensive drugs have differential effects on brachial and central pressure ^(36)^. In 2013, hypertension guidelines have added aortic PWV measured by carotid-femoral PWV (cf-PWV) as a recommended test for the detection of large artery stiffening associated with cardiovascular disease ^(37)^.

### Heart failure

Patients with heart failure have lower PWV than normal due to reduced stroke volume and BP when the heart fails to pump adequate blood flow through the circulatory system and organs ^(38)^. It has been demonstrated that in the process of heart failure, an increase in peripheral vascular resistance resulting in the rise in aortic wave reflections has adverse effects on increased ventricular afterload, including decreased coronary perfusion ^(18)^. Moreover, increased central arterial wave reflection detected at the carotid artery in the early stage of heart failure has been shown to independently predict cardiovascular risk and mortality ^(39)^.

### Stroke

Early detection and effective control of risk factors of atherosclerosis can significantly reduce the possibility of stroke. For example, high BP is a major cause of stroke, and reduced BP can significantly reduce the risk of stroke. Thus, central pressure monitoring at the carotid artery is better related to the risk of stroke and can be controlled by anti-hypertensive medications. PWV and flow velocity at the carotid bifurcation are routinely checked at the neck *via* Doppler ultrasound for the high risk population. Aortic PWV is considered a powerful stroke risk indicator in hypertensive patients, and its integration into clinical follow-up programs in patients with CVD risk is recommended ^(40)^. In addition, an evidence shows that aortic PWV (cf-PWV) in an increment of 1 m/s can increase the risk of stroke by 40% ^(40)^.

Clinical evidence from the Framingham Study involving middle-aged and older people with 2,232 participants reported that aortic PWV was associated with a 48% increase in CVD risk, whereas central pulse pressure, augmentation index, and carotid-brachial pressure amplification were not associated with CVD events ^(41)^. Measures of PWA are associated with CVD risk factors and target organ damage due to increased arterial stiffness and impaired arterial elastic properties ^(42)^. Table 1 presents a variety of example studies in the relation between CVDs and carotid PWA.

## PWA and Noninvasive Blood Pressure Estimation

Researchers have investigated the link between the PWA character and noninvasive BP estimation. The augmentation of the systolic and diastolic pressure due to the reflected wave confirmed that PWA may be used for noninvasive BP analysis ^(58)^. Several studies have discussed cuffless BP estimation using photoplethysmogram (PPG) signals based on optical sensor ^(59)^ and pressure sensing technique measuring a pressure pulse wave (PPW) with a pressure sensor. The process of calculating the changes in light absorption from PPG waveforms induced by BP may be unstable, unreliable, and interfered by motion artifact, respiration effect, and low perfusion ^(60)^. The pressure sensing technique that reflects the pressure changes within the blood vessel in the artery has less interference and more abundant characteristic information ^(61)^. PPW can be collected in a wearable manner with the development of electric fabric and flexible pressure sensors ^(62)^, which makes the utilization of PPW for cuffless and wearable BP estimation more feasible. The use of a hemodynamic wearable technology with e-Health may allow the creation of registries and collection of big data, which is useful for the implementation of artificial intelligence (AI) and machine learning (ML) algorithms so as to predict the cardiovascular risk and act as a guide in clinical management ^(63)^. Furthermore, incorporating the data-driven algorithms of deep learning can target information on the effects of interventions, including pharmacological therapy, lifestyle modifications, and supplemental materials on PWA in association with vascular function and future cardiovascular events for individual assessment and personalized medicine.

## Future for PWA of the Carotid Artery

**Figure 4.**
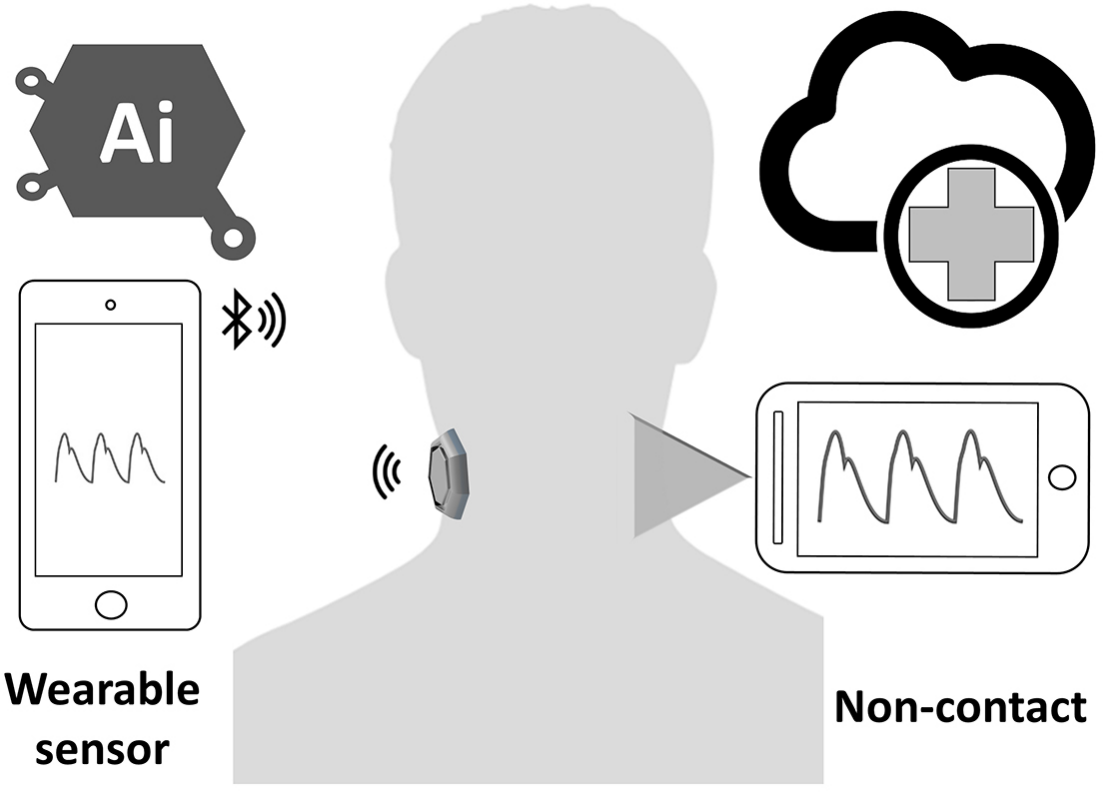
Wearable and noncontact sensors for measuring carotid pulse wave using e-Health system.

Functional impairment of the carotid arterial wall may occur before the development of structural wall changes. Any detection methods of arterial function regarding the PWA changes can identify the early stage of the atherosclerotic process before the occurrence of clinical symptoms of cardiovascular diseases. Numerous studies evaluated the PWA of the arteries using carotid tonometry. However, this technique is currently less preferred for pulse pressure analysis. It is difficult to maintain adequate stabilization and applanation because the artery can be moved easily under the sensor; hence, a professional operator is required ^(64)^. Therefore, a new technology of wearable sensors (Figure 4) is well suited for replacing traditional tonometry settings with various types of sensors, such as flexible pressure sensor produced from polydimethylsiloxane ^(65)^, bandage-like flexible pressure sensor ^(66)^, fingerprint-like ferroelectric films ^(67)^, ultrathin inorganic piezoelectric ^(68)^, droplet-based pressure sensor ^(69)^, accelerometric sensors ^(70)^, and bidirectional microphone ^(71)^. The development of successful devices that use a carotid wearable sensor may have a large potential of clinical applications over the traditional cuff BP. A number of researchers proposed noncontact techniques for the measurement of PWV and pulse waveform at the carotid artery, including interferometry coupled with fiber optics ^(72)^, laser Doppler vibrometer (LDV) ^(73)^, and optical vibrocardiography ^(74)^. However, these noncontact methods require a complicated setup with cumbersome devices that are not currently available at a clinical setting.

## Conclusion

Over the past several years, numerous clinical studies and meta-analyses have demonstrated that PWA associated with arterial stiffness is an independent predictor of cardiovascular events. Indeed, carotid PWA and PWV as measures of arterial stiffness are more predictive of CVD risk and provide the prognostic advantage over brachial artery pressure ^(75)^. The measurement of PWV, which is simple, noninvasive, and reproducible, may be a useful tool to select subjects who are at a high risk of developing subclinical atherosclerosis or CVD, especially in mass screening. This review presents the general overview, potential utilization, and examples of clinical measurements of PWA reflecting the status of the cardiovascular system. This evidence demonstrates a high potential for the application of carotid pulse measurement as a better biomarker for CVD risk evaluation and hemodynamic monitoring with clinical research studies using advanced wearable sensors developed during the era of precision medicine.

## Article Information

### Conflicts of Interest

None

### Author Contributions

SP reviewed the literature and drafted the manuscript. All authors contributed to the concept of the manuscript, interpretation of the literature, and critical review of the manuscript and approved the final version.

### Approval by Institutional Review Board (IRB)

Not applicable.
